# Uptake of the Second Dose of the Measles Vaccine and Its Determinants Among Children Aged Less Than 5 Years: Systematic Review and Meta-Analysis

**DOI:** 10.2196/77195

**Published:** 2025-08-27

**Authors:** Molalign Aligaz Adisu, Tesfaye Engdaw Habtie, Tegene Atamenta Kitaw, Alemu Birara Zemariam

**Affiliations:** 1 Department of Pediatrics and Child Health College of Health Science Woldia University Woldia Ethiopia; 2 Department of Nursing College of Health Science Woldia University Woldia Ethiopia

**Keywords:** second dose, measles, vaccine, uptake, children, Ethiopia, meta-analysis

## Abstract

**Background:**

Ethiopia is faced with poor measles-containing vaccine second dose (MCV2) coverage, leading to recurrent outbreaks.

**Objective:**

This meta-analysis and systematic review aimed at combining evidence of MCV2 uptake in Ethiopia and its determinants to inform interventions for increased vaccination uptake and control of public health challenges.

**Methods:**

This review examined observational quantitative research on measles second dose vaccination among children in Ethiopia using databases such as PubMed Central, Cochrane Library, Web of Science, Scopus, MEDLINE, and Embase. The quality of the included studies was assessed using the Newcastle-Ottawa Scale. Heterogeneity was evaluated using *I*^2^ statistics and the Cochran *Q* test, and the analysis used a random-effects model. Publication bias was assessed through funnel plots, the Egger test, and nonparametric trim-and-fill tests.

**Results:**

The overall MCV2 uptake among children aged <5 years in Ethiopia was only 34.4% (95% CI 18.8%-49.9%). Significant determinants of MCV2 uptake included high level of maternal education (adjusted odds ratio [AOR] 3.31, 95% CI 1.32-5.30), attendance to antenatal care follow-ups (AOR 2.02, 95% CI 1.12-2.92), use of postnatal care services (AOR 3.03, 95% CI 1.77-4.28), reduced waiting times at vaccination sites (AOR 2.56, 95% CI 1.98-3.13), good awareness of measles vaccination (AOR 2.17, 95% CI 1.59-2.74), and positive perceptions of the vaccine (AOR 3.58, 95% CI 1.97-6.30).

**Conclusions:**

This study found that the uptake of MCV2 among children aged <5 years in Ethiopia was 34%, which is far below the global and national goal of 95%. Key factors contributing to low coverage include mothers’ educational levels, use of antenatal and postnatal care services, waiting times at vaccination sites, and mothers’ awareness of the measles vaccine. Improving community-based education programs, increasing access to antenatal and postnatal care services, reducing waiting times, and raising awareness about immunization all contribute to increasing vaccine uptake.

**Trial Registration:**

PROSPERO CRD42024619031; https://www.crd.york.ac.uk/PROSPERO/view/CRD42024619031

## Introduction

### Background

Measles, a virulent viral disease caused by the measles morbillivirus, continues to exert a significant global health burden despite decades of vaccination efforts. The disease is characterized by fever, maculopapular rash, cough, coryza, and conjunctivitis. Alongside these symptoms, severe complications such as encephalitis, severe pneumonia, and even death may occur. These complications tend to be worse in young children, malnourished individuals, and people with a weakened immune system [1,2]. The highly contagious nature of measles is further highlighted by its basic reproductive number (R0), which is estimated to be between 12 and 18. This elevates the threshold population immunity required to prevent sustained transmission of the virus to >95% immunization [3].

The lack of global progress made toward measles control in the early 21st century is concerning, especially with the recent regressions observed. One study revealed the increasing rate of measles infections worldwide, projecting that 10.3 million people were infected in 2023, which is a shocking 20% increase from the previous year’s estimate. Moreover, the number of countries with outbreaks also increased from 36 to 57 [4].

This surge critically underscores the precarious state of global immunity, a condition made even more fragile by disruptions to routine immunization services—such as those observed during the COVID-19 pandemic—and compounded by prevailing vaccine hesitancy across many populations [5,6]. In fact, insufficient coverage of immunization is the main impetus for these increasing outbreaks. More than 22 million children worldwide missed their first dose of the measles vaccine in 2023 alone—this creates growing susceptible cohorts that fuel epidemics [5].

The burden of measles falls on low- and middle-income countries. That is where health systems become even more strained, in many cases due to infrastructural, financial, and geographical barriers preventing access to strong equitable routine immunization services [7]. Measles can certainly be a disaster for morbidity and mortality within vulnerable populations but also severely strain already weak health care infrastructures by taking away badly needed resources from other important frontlines of public health, as well as increasing existing inequities in these vulnerable populations. The big picture goal of global measles elimination—as so unmistakably articulated in the World Health Organization (WHO) Immunization Agenda 2030—is decidedly imperiled at present by exactly such gaps in coverage, thus demanding recommitment and inspiring novelty in contextualized strategies directed toward achieving the lofty coverage rates necessary for eradication [8-10].

Prevention of measles and its complications is best achieved through vaccination. There are several live, attenuated measles vaccines that range from monovalent vaccines to combinations under the “measles-containing vaccine” name that also provide immunity against rubella, mumps, and varicella. Both doses of the measles vaccine currently in use worldwide are safe and effective. The first dose of the measles-containing vaccine (MCV1) was introduced in 1963, and by the 1980s, the WHO recommended that all countries implement routine MCV1 vaccination in their childhood immunization schedules. Ethiopia followed suit and introduced the MCV1 in the late 1980s [11,12].

Even following the introduction of the MCV1, which provides immunity to 85% of vaccine recipients, up to 15% of individuals do not develop long-term immunity following a single dose. MCV1 in some cases will also not generate an adequate immune response to provide immunity, a condition that comes to be known as primary vaccine failure. Identifying this gap, the WHO recommended in 2009 that countries introduce a second dose of the measles-containing vaccine (MCV2) into the national immunization schedules after they achieved 80% MCV1 uptake for 3 consecutive years. However, in 2017, the WHO revised this and recommended the introduction of MCV2 into national immunization schedules regardless of the level of uptake of MCV1 [13,14]. In response, Ethiopia introduced the MCV2 in February 2019 [15].

To maximize the effectiveness of vaccination, WHO guidelines stipulate that, in regions where the risk of measles mortality among infants is high, the MCV1 should be administered at 9 months of age followed by the MCV2 at the ages of 15 to 18 months. In lower-risk countries, the MCV1 may be administered at 12 months, with MCV2 timing adjusted to achieve the highest uptake by children [13]. Given the high risk of measles mortality in Ethiopia, both doses are administered at 9 and 15 months, respectively [16-18].

Even with national efforts and strategic frameworks adopted by the Ethiopian government as part of measles elimination efforts by 2030—strengthening routine immunization services, supplementary immunization activities, and surveillance—there remains a profound burden of measles in Ethiopia. Recent data clearly show an alarming reality that underscores gaps left behind in immunization coverage [19]. The United Nations Children’s Fund and Centers for Disease Control and Prevention reports place Ethiopia consistently among those countries that have a large number of unvaccinated children [20,21]. Furthermore, it has always been among the topmost countries reporting measles cases, with both periodic and significant outbreaks across its regions [21].

Recent studies on measles epidemics in Ethiopia have described the entire picture of systemic and operational factors that contribute such challenges. A detailed review of outbreak investigations carried out in 2024 highlighted critical issues such as staff absenteeism, vaccines being out of stock at different levels of the supply chain, and shortage of operational funding leading to high dropout rates in vaccination and compromised service delivery [22]. Studies carried out in 2023 and 2024 further detailed such barriers from a qualitative perspective by introducing community-based factors—rural residence; low educational level of mothers among their sociodemographic characteristics; perceived safety of vaccines; inadequate health facilities within accessible distance; and inadequate community mobilization that impedes attainment of adequate coverage, particularly for the second dose [23,24]. Consequently, MCV2 uptake in Ethiopia persistently remains below both national targets and the critical 95% threshold necessary for achieving population-level herd immunity, contributing directly to the ongoing susceptibility of communities and the perpetuation of the disease [18,25]. Therefore, addressing this *last mile* gap in MCV2 coverage is identified as an urgent public health priority.

### Objectives

Even though individual studies have looked at different aspects of vaccine acceptance and uptake across various regions of Ethiopia, there is still a lack of clear, comprehensive, and quantitative insights into the specific factors influencing MCV2 uptake at the national level. This systematic review and meta-analysis aimed to bring together all available data from Ethiopia on MCV2 uptake and its key determinants. By thoroughly synthesizing the existing evidence, we aimed to deepen the understanding of this pressing public health issue and support the development of more effective, evidence-based strategies to protect the health of Ethiopian children—and, ultimately, help achieve global and national measles elimination goals.

## Methods

### Study Design and Reporting

This systematic review and meta-analysis aimed to provide an estimate of the overall MCV2 uptake and determinants among children aged <5 years in Ethiopia. This review used the PRISMA (Preferred Reporting Items for Systematic Reviews and Meta-Analyses) guidelines. In addition, this review has been registered with PROSPERO with protocol CRD42024619031.

### Inclusion and Exclusion Criteria

The inclusion and exclusion criteria for this review can be found in Textbox 1.

**Inclusion and exclusion criteria for the systematic review and meta-analysis on the uptake of the second dose of the measles vaccine and its determinants among children aged <5 years in Ethiopia (criteria defined for study selection and rationale; 2025).**

**Inclusion criteria**

- Setting: research conducted only in Ethiopia
- Population: studies involving children
- Language: published in English
- Study design: observational studies (longitudinal, cohort, cross-sectional, or case-control)
- Outcome variables: clearly defined outcome variables
- Quality: studies of sufficient quality
- Access: accessible through electronic resources

**Exclusion criteria**

- Setting: studies conducted outside of Ethiopia
- Population: studies focusing on adults or other populations
- Language: published in languages other than English
- Study design: experimental studies or reviews
- Outcome variables: undefined or unclear outcome variables
- Quality: low-quality studies
- Access: studies for which necessary information could not be obtained due to unavailability of the full text

### Search Strategy and Source of Information

The search strategy was built using an adapted population, exposure, outcomes, study design, and setting framework to generate the MeSH (Medical Subject Headings) terms necessary to search for appropriate studies in the databases as follows:

1. Population: “children” and “pediatrics population”
2. Exposure: “second-dose measles vaccine” and “second-dose measles-containing vaccine”
3. Outcome: “uptake,” “utilization,” “coverage,” “associated factors,” “predictors,” “barriers,” or “determinants”
4. Study design: “observational studies”
5. Setting (context): “Ethiopia”

For a systematic review with primary studies as part of these studies, review questions focused on Ethiopia based on the aforementioned format were as follows: (1) what is the aggregate uptake of MCV2 among children aged <5 years in Ethiopia? (2) What associated factor explains the uptake of the MCV2 among children in Ethiopia?

A systematic search was conducted in various electronic databases: PubMed Central, Cochrane Library, Web of Science, Scopus, MEDLINE, Embase, and Google Scholar. The search used specific free-text terms and Boolean operator strings to locate studies pertinent to the aforementioned questions: (“Second-dose measles vaccine” OR “Second-doses measles-containing vaccine” OR “two-doses of measles vaccine” OR “measles vaccination”) AND (“children” OR “pediatrics”) AND (“uptake” OR “utilization” OR “coverage” OR “acceptance” OR “adherence”) AND (“associated factors” OR “predictors” OR “barriers” OR “determinants” OR “influencers” OR “challenges”) AND (“Ethiopia” OR “Ethiopian context” OR “Ethiopian children”). After retrieving accessible articles, all the results were sorted, and duplicates were removed.

### Study Selection and Quality Assessment

Two investigators, TEH and TAK, conducted a preliminary screening of studies by reviewing titles and abstracts to identify potentially relevant research before retrieving the full texts. If additional clarification was required to determine eligibility, they reached out to the corresponding author (MAA). Any disagreements between the investigators were resolved through discussion to achieve a consensus on inclusion or exclusion. All retrieved articles were imported into EndNote (version 20; Clarivate Analytics), where duplicate entries were eliminated. Next, the researchers reviewed the articles independently based on their structures and purposes, searching titles, abstracts, and full texts for studies that met the predetermined inclusion criteria. The articles for this review were selected and analyzed jointly by both reviewers.

To ensure the quality of each study, the adapted Newcastle-Ottawa Scale was used, which was reviewed by 3 independent assessors. The assessors undertook an inclusive review of all articles that met the inclusion criteria and study purpose. Differences between the reviewers were resolved through consensus, and a fourth reviewer was involved in adjudication as necessary.

The criteria for the assessment addressed a series of key areas: representativeness of the sample, adequacy of the sample size, response rates, respondent and nonrespondent characteristics, quality of the intended measurement tools for exposure or risk factors, comparability of outcome groups based on study design, controlling for major confounding variables, and measurement of outcome and use of appropriate statistical tests. Each of the studies was scored out of 10 points, with scores of >7 indicating low risk of bias for inclusion. All studies included in the analysis had a low risk of bias, with scores ranging from 9 to 10 (the full Newcastle-Ottawa Scale results can be found in Table S1 in Multimedia Appendix 1 [26-36]).

### Data Extraction

The data extraction form was developed using a Microsoft Excel spreadsheet. Two independent reviewers (MAA and ABZ) systematically extracted data from the full-text articles. The data extraction form included key variables such as the first author’s name, year of publication, study region, study design, sample size, age of the participants, sample distribution by sex, uptake or coverage, and the number of children vaccinated with MCV2.

For each primary outcome, the uptake of MCV2 was recorded, whereas adjusted odds ratios (AORs) for each associated factor (secondary outcome) were also noted along with 95% CIs. Any discrepancies between the data extractors were addressed through discussion and consensus with another author.

### Statistical Analysis

After extracting the data, a meta-analysis was conducted using Stata (version 17; StataCorp). Uptake estimates were calculated along with their corresponding SEs using the formulas *p* = *r*/*n* and SE = √*p*(1 − *p*)/*n*, where *p* represents the proportion, *r* is the total number of children vaccinated with MCV2, and *n* is the sample size. The results of the meta-analysis are presented as the pooled uptake of MCV2 accompanied by 95% CIs. A significance threshold was established at *P* values of <.05.

To investigate the factors influencing the uptake of MCV2, Stata (version 17) was used. A random-effects model was applied as heterogeneity between studies was high, as confirmed through an *I*^2^ statistic of >75% and a *P* value of <.05. The *I*^2^ index and Cochran *Q* test were used for testing heterogeneity. The *I*^2^ statistic ranges from 0% to 100%, with 0% indicating no heterogeneity and 100% indicating high heterogeneity.

The effect of individual studies on the overall prevalence estimate was tested using sensitivity analysis. Publication bias was also tested using funnel plots for symmetry assessment, as well as the Egger test and nonparametric trim-and-fill tests. Although both these tests were used, the Egger test is commonly deemed superior for detecting publication bias because it provides actual effect sizes along with their accuracy. Contrary to this, the funnel plot is a less objective measure of potential asymmetry and may hint at publication bias.

## Results

### Literature Search

**Figure 1:**
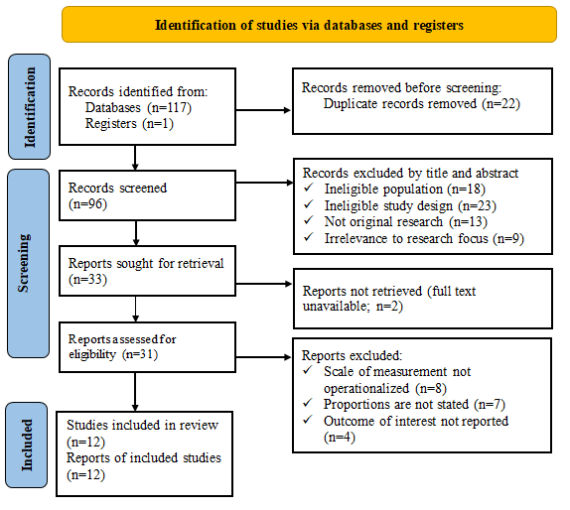
Identification of studies via databases and registers.

This systematic review and meta-analysis followed the PRISMA guidelines (Multimedia Appendix 2). A comprehensive search of PubMed Central, Cochrane Library, Web of Science, Scopus, MEDLINE, and Embase initially yielded 118 articles addressing MCV2 uptake and its predictors among Ethiopian children. Of these 118 articles, after removing 22 (18.6%) duplicate records, 96 (81.4%) underwent title and abstract screening. Of these 96 articles, 63 (66%) were excluded for the following reasons: ineligible population (n=18, 29%), ineligible study design (n=23, 37%), nonoriginal research (n=13, 21%), or irrelevance to the research focus (n=9, 14%). The remaining 33 full-text articles were assessed for retrieval, with 2 (6%) records unavailable. Of the 31 articles evaluated in full text, 19 (61%) were excluded due to non-operationalized measurement scales (n=8, 42%), unstated proportions (n=7, 37%), or unreported outcomes of interest (n=4, 21%). Ultimately, 12 studies met all the inclusion criteria and were included in the final analysis (Figure 1).

### Study Characteristics

The total review comprised 12 studies on 7847 children from all regions of the country. Specifically, the proportion of studies by region was 50% (6/12) of the studies conducted in Amhara, 25% (3/12) in Oromia, 8% (1/12) in Somalia, and 17% (2/12) from all regions. A total of 92% (11/12) of the studies were cross-sectional, and 8% (1/12) were case-control studies. All studies (12/12, 100%) were conducted during the period from 2019 to 2023. The average number of participants per study was 654 (SD 254; range 372-1172). Almost half (49%) of the participants were female (Table 1).

**Table 1:** Descriptive summary of the studies analyzed in the meta-analysis on measles vaccine second dose uptake and its determinants among children aged <5 years in Ethiopia (including authors, study design, participant ages, sample size, locations, and key findings).

Study	Year conducted	Region	Design	Participant age (y)	Sample size, n	Male participants, n	Female participants, n	Prevalence, %	Outcome
Taffie et al [26]	2022	Amhara	CS^a^	24-35	418	192	226	41.39	173
Tadesse et al [27]	2022	Oromia	CS	15-36	372	—^b^	—	42.5	158
Adisu et al [28]	2023	Amhara	CS	24-36	621	319	302	75.68	470
Demewoz et al [29]	2020	Amhara	CS	24-35	837	406	431	48.1	403
Woyessa et al [23]	2021	Oromia	CS	24-35	1172	558	613	48	563
Goshu Muluneh et al [30]	2019	All regions	CS	12-36	965	545	420	12.36	119
Ibrahim et al [31]	2023	Somalia	CS	15-36	429	215	214	21.4	92
Nurgi et al [32]	2023	Oromia	CC^c^	18-24	446	—	—	75.3	336
Teshale and Amare [33]	2019	All regions	CS	24-35	800	424	376	9.84	79
Zeleke et al [34]	2022	Amhara	CS	15-23	633	332	301	53.08	336
Mulatu et al [35]	2022	Amhara	CS	24-35	732	390	342	63.3	463
Asichalew [36]	2021	Amhara	CS	24-35	422	199	223	53	224

^a^CS: cross-sectional.

^b^Not available.

^c^CC: case-control.

### Publication Bias

**Figure 2:**
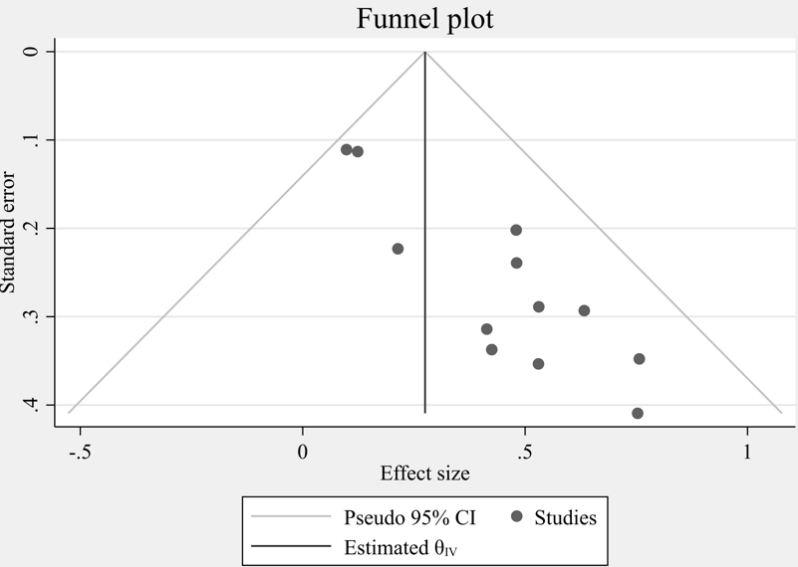
Funnel plot illustrating publication bias for second-dose measles vaccine uptake among children younger than 5 years in Ethiopia (analysis conducted in 2025), reflecting study distribution and variability.

The publication bias was estimated through funnel plot analysis and the Egger regression test. The funnel plot revealed an asymmetrical shape, intuitively suggesting the presence of publication bias (Figure 2). The Egger regression test also yielded a *P* value of .001, indicating statistical evidence of publication bias. This agreement between subjective and objective analysis strengthens the likelihood of bias in the published studies.

To explain the potential issue of publication bias indicated by the asymmetrical funnel plot, a nonparametric trim-and-fill analysis was conducted to calculate the overall effect. The trim-and-fill analysis flagged 18 studies and imputed 6 studies to calculate the potential bias. The observed effect size was 34.4 (95% CI 18.8-49.9). Following imputation of the studies, the effect size was 20.6 and was well within the CI of the observed effect. This means that the imputed studies had zero or negligible contribution to the overall effect size. The trim-and-fill analysis was used to include any missing studies due to publication bias and provide a better estimate of the true effect size. The tiny difference between the observed and adjusted effect sizes informed us that publication bias was likely not a significant problem in this dataset.

### Pooled Uptake of MCV2 Among Children in Ethiopia

**Figure 3:**
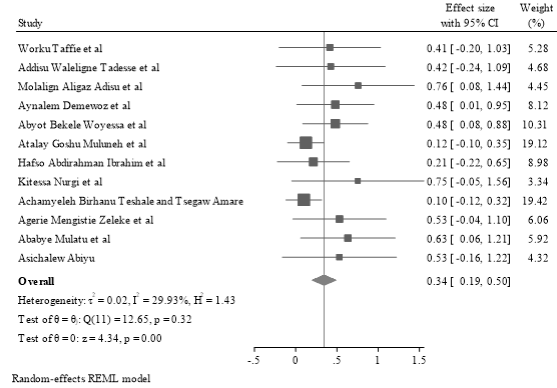
Forest plot depicting the pooled uptake of second-dose measles vaccine among children younger than 5 years in Ethiopia.

The pooled uptake of MCV2 among children in Ethiopia was 34.4% (95% CI 18.8%-49.9%), and there was low heterogeneity between the studies (*P*=.32; τ^2^=0.02; *I*^2^=29.9%; *H*=1.41; Figure 3 [26-36]).

### Sensitivity Analysis

**Figure 4:**
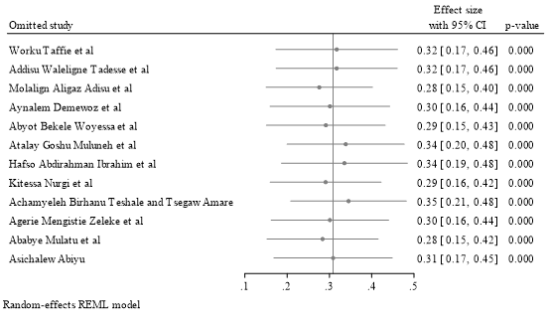
Results of the sensitivity analysis on the uptake of second-dose measles vaccine among under-five children in Ethiopia.

Sensitivity analysis was conducted to evaluate the influence of outlying or potentially influential studies on the overall uptake estimate of the MCV2 among children. Through a random-effects model, the results of this analysis showed that there were no influential studies or outliers detected because all the point estimates fell within the limits of the 95% CI. This finding indicates that the overall estimate of uptake was robust and not determined to a large degree by a single study, thus providing additional strength for the validity of the pooled results. The full sensitivity analysis results are presented in Figure 4 [26-36].

### Factors Associated With the Uptake of MCV2 in Ethiopia

The pooled odds ratio for mothers with the highest educational attainment as indicated in the studies was 3.31 times greater for vaccinating their children with MCV2 than for mothers with low or no education (AOR 3.31, 95% CI 1.32-5.30). Moreover, mothers who attended antenatal care (ANC) follow-ups were over twice as likely to vaccinate their children with MCV2 compared to those who did not attend ANC follow-ups (AOR 2.02, 95% CI 1.12-2.92). Similarly, mothers who used postnatal care (PNC) services were 3 times more likely to vaccinate their children with MCV2 than mothers who did not use PNC services (AOR 3.03, 95% CI 1.77-4.28).

In addition, mothers who had a waiting time of <30 minutes at vaccination centers were 2.5 times more likely to vaccinate their children than those with waiting times of >30 minutes (AOR 2.56, 95% CI 1.98-3.13). Moreover, mothers who were highly aware of measles vaccination were twice as likely to vaccinate their children with the second dose as mothers who were lowly aware (AOR 2.17, 95% CI 1.59-2.74). Similarly, the odds of being vaccinated with the MCV2 were 3.5 times higher (AOR 3.58, 95% CI 1.97-6.30) among children of mothers who had a positive perception of the measles vaccination compared to those of mothers with a poor perception (Table 2).

**Table 2:** Pooled estimates of determinants affecting the uptake of the second dose of the measles vaccine among children aged <5 years in Ethiopia (analyzed in 2025).

Associated factors	Pooled effect size (95% CI)	*I*^2^ (%)	Egger test *P* value	Studies pooled
Mothers who attained the highest level of education	3.31 (1.32-5.30)	0	—^a^	[23,31]
Mothers who attended ANC^b^ service follow-up	2.02 (1.12-2.92)	0	.40	[28,34]
Mothers who attended PNC^c^ service follow-up	3.03 (1.77-4.28)	18.64	.19	[26,28,31,32]
Waiting time of <30 min at the vaccination site	2.56 (1.98-3.13)	0	.62	[27,34]
Mothers who had good awareness	2.17 (1.59-2.74)	0	.01	[26-29,31]
Mothers who had a positive perception	4.30 (2.63-5.97)	0	.35	[28,29]

^a^Not available.

^b^ANC: antenatal care.

^c^PNC: postnatal care.

## Discussion

### Principal Findings

Pooled uptake of MCV2 among children aged <5 years in Ethiopia was 34.4% (95% CI 18.8%-49.9%). This result is in line with the statistics from Nigeria, Madagascar, and Afghanistan, where low uptake rates have been documented [37-39]. However, this figure is significantly lower than the WHO target of 95% for MCV2 uptake, as well as the national immunization targets set by Ethiopia and other countries [38,40-42]. A WHO report showed that there are countries even below this level of uptake, such as the Democratic Republic of the Congo, Uganda, and Angola [38], which poses a substantial risk to public health in these regions. Several factors contribute to the low vaccination rates in Ethiopia. Ongoing strife and instability in certain regions have significantly hampered health care provision and access to vaccine services. Internal displacement and conflict have led to destruction of health infrastructure, which is making it increasingly difficult for families to access core immunization services. In addition, socioeconomic obstacles for several families, such as illiteracy and poverty, further compound the problem, leading to poor awareness and prioritization of vaccination. The consequences of the very low uptake are extensive. A high level of vaccination uptake should be reached if measles epidemics are to be avoided and herd immunity is to be achieved. All this emphasizes once more the imperative of urgent specific interventions aimed at improving vaccine uptake. Some likely options include increasing public awareness regarding the importance of the second dose, enhanced health service access, and targeting obstacles keeping families from vaccinating their children. Without significant improvements in these areas, the goal of measles elimination in Ethiopia and similar contexts will remain unattainable.

The pooled odds ratio indicated that mothers with the highest educational attainment were 3.31 times more likely to vaccinate their children with MCV2 compared to mothers with low or no education. Similar observations have been recorded in other sub-Saharan countries [41,43]. This is because those mothers who are better educated to some level will understand the benefit of vaccination, appreciate the hazard of vaccine-preventable diseases, and be in a position to avail their children care. This underscores the value of targeted educational interventions to increase awareness and knowledge of the importance of immunization, particularly among more poorly educated communities. Through investment in education, public health interventions will be able to improve levels of vaccination uptake and, ultimately, child health outcomes among high-risk groups.

The findings indicate that maternal attendance to ANC and PNC services significantly influences whether children are immunized with MCV2. Specifically, mothers who attended ANC follow-ups had twice the chances of vaccinating their children compared to those who did not attend ANC follow-ups. Similarly, mothers using PNC services were 3 times more likely to vaccinate their children with MCV2 than those who did not receive PNC services. Similar results have been reported in sub-Saharan African and South Asian countries [41,44]. This is because women attending ANC and PNC are most likely to know more about health habits, including the significance of vaccinations. During these visits to health care facilities, there are opportunities for health care workers to inform mothers of the advantages of vaccination, clarify queries, and provide support for appropriate timing of vaccination. All these findings demonstrate the importance of maternal health care uptake in the initiation of immunization.

The combined outcome revealed that mothers who waited for <30 minutes at the vaccine center were 2.5 times more likely to vaccinate their children compared to those waiting for >30 minutes. This is consistent with findings reported in other studies [45]. This is because shorter waiting times enhance perceived convenience in attending vaccination sessions. When mothers enjoy a smooth experience, they tend to perceive vaccination as possible and, hence, have a high compliance. In contrast, longer waiting times provide opportunities for mothers to become exhausted and stressed, especially with respect to children, who are most likely to become restless. Reduced waiting times create a smoother experience, allowing mothers to proceed with vaccinations easily. Apart from this, time is a valuable resource for most families, with women typically having a work schedule as well as caring duties. Thus, it is preferable to minimize waits to counter the opportunity cost of attending vaccinations. This provides evidence of the significant impacts of waiting times on acceptance of vaccinations, suggesting the role of the efficiency of the vaccination process in shaping immunization uptake.

The combined findings indicate that maternal perception and awareness of measles vaccination exert a significant influence on children vaccination uptake. Mothers with high awareness of measles vaccination were more than twice as likely to vaccinate their children with the second dose compared with mothers with low awareness. In addition, the odds of vaccinating children with the second dose were 3.5 times higher among mothers with a positive perception of measles vaccination. This outcome is consistent with those of a study conducted in sub-Saharan Africa [43,46]. This is because educated mothers who comprehend the value and advantages of measles vaccination are most likely to place greater emphasis on immunization among their children. Effective awareness helps in clearing misconceptions and myths that could lead to vaccine hesitation [46,47]. Moreover, when mothers believe in the efficacy and safety of vaccines, they are more likely to adhere to immunization schedules. Such beliefs may be based on personal experience, social norms, or successful health promotion. These findings highlight the exceptional significance of enhancing maternal attitudes and perceptions regarding measles vaccination. Vaccine benefit knowledge can significantly boost rates of vaccination among children. Public health interventions should be grounded in educational activities and community mobilization to establish an enabling environment for immunization.

This study has several limitations that warrant acknowledgment. First, the small sample size and cross-sectional design restrict both statistical power and the ability to infer causality or temporal relationships concerning vaccine acceptance. A significant limitation stems from the potential publication bias observed among the included studies (Egger test: *P*=.001), which, despite mitigation efforts such as trim-and-fill analysis, could still influence the pooled estimates. This is compounded by the reliance on self-reported data within many of the included studies, introducing potential social desirability and recall biases, particularly for subjective measures such as awareness. Furthermore, there was a notable geographical imbalance, with a disproportionate number of included studies originating from the Amhara region and underrepresentation of other diverse Ethiopian contexts. This limits the generalizability of our findings across the nation’s varied cultural and health care landscapes. Finally, the analysis did not fully account for the impact of temporal factors and policy changes, especially given the introduction of MCV2 in Ethiopia in 2019 and the varying time frames of the included studies. Important covariates such as cultural beliefs; accessibility challenges (eg, rural vs urban disparities); and conflict-related disruptions, which are known to influence vaccination rates in Ethiopia, were also not explicitly explored. Future research should aim to address these gaps to provide a more comprehensive understanding of vaccine uptake determinants.

### Conclusions and Recommendations

The pooled MCV2 uptake among Ethiopian children aged <5 years was 34%, which is far below the national target of 95%. The level of education of mothers, use of ANC and PNC services, waiting time at vaccination sites, and mothers’ awareness and perceptions of measles vaccination were statistically significant predictors of uptake of MCV2. To increase the uptake of MCV2 in Ethiopia, it is necessary to target interventions. Interventions should target the strengthening of community-based educational programs, mobile clinics for remote areas, enhanced access to ANC and PNC services, reduction of waiting times at the point of vaccine delivery, and raising awareness about the benefits of measles vaccination. These multistakeholder initiatives are crucial in achieving the national immunization objective.

## Appendix

Multimedia Appendix 1 Newcastle-Ottawa Scale scores for included studies.

Multimedia Appendix 2 PRISMA 2020 Checklist.

## Abbreviations

ANC: antenatal care
AOR: adjusted odds ratio
MCV1: first dose of the measles-containing vaccine
MCV2: second dose of the measles-containing vaccine
MeSH: Medical Subject Headings
PNC: postnatal care
PRISMA: Preferred Reporting Items for Systematic Reviews and Meta-Analyses
WHO: World Health Organization

## Data Availability

The datasets generated or analyzed during this study are available from the corresponding author (MAA) on reasonable request.
