# Supplementary material for: Uptake of the Second Dose of the Measles Vaccine and Its Determinants Among Children Aged Less Than 5 Years: Systematic Review and Meta-Analysis
Source: JMIR Public Health Surveill. 2025 Aug 27;11:e77195. doi: 10.2196/77195 (PMC12423608; doi:10.2196/77195)
Supplement: Multimedia Appendix 1 [file publichealth_v11i1e77195_app1.pdf]

Supplemental table 1: Quality assessment included studies using modified Newcastle - Ottawa Scale (NOS)

**First author, study year**	**Criteria**							
	**Selection**				**Comparability**	**Outcome**		
	Representativeness of the sample	Sample size	Non – respondents	Ascertainment of exposure/risk factor	Comparability of subjects in different outcome groups on the basis of design or analysis. Confounding factors controlled.	Assessment of the outcome	Statistical test	**Total score (10)**
Worku Taffie et al (2022)	1	1	1	2	2	2	1	**10**
Addisu Waleligne Tadesse et al (2022)	1	0	1	2	2	2	1	**9**
Molalign Aligaz Adisu et al (2023)	1	1	1	2	1	2	1	**10**
Aynalem Demewoz et al (2020)	1	1	1	2	2	2	1	**10**
Abyot Bekele Woyessa et al (2021)	1		1	1	2	2	1	**9**
Atalay Goshu Muluneh et al (2019)	1	1	1	2	2	2	1	**10**
Hafso Abdirahman Ibrahim et al (2023)	1	1	1	2	2	2	1	**10**
Kitessa Nurgi et al (2023)	1	1	1	2	2	2	1	**10**
Achamyeleh Birhanu and Tsegaw Amare (2019)	1	1	1	2	2	2	1	**10**
Agerie Mengistie Zeleke et al (2022)	1	1	1	1	2	2	1	**10**
Ababye Mulatu et al (2022)	1	1	1	2	2	2	1	**10**

Asichalew Abiyu (2021)	1	1	1	2	2	2	1	**10**

Score (Stars)	Quality Classification	Interpretation
**≥7**	**High quality**	Low risk of bias; results are reliable.
**5–6**	**Moderate quality**	Some risk of bias; interpret with caution.
**≤4**	**Low quality**	High risk of bias; findings may be unreliable

NEWCASTLE - OTTAWA QUALITY ASSESSMENT SCALE

Selection: (Maximum 5 stars)

1) Representativeness of the sample:

a) Truly representative of the average in the target population (all subjects or random sampling). 1 score
b) Somewhat representative of the average in the target population (Nonrandom sampling). 1 score
c) Selected demographic group of users. 0 score
d) No description of the sampling strategy. 0 score

2) Sample size:

a) Justified and satisfactory (≥ 400 included). 1 score
b) Not justified (< 400 included). 0 score

3) Non-respondents:

a) Proportion of target sample recruited attains pre-specified target or basic summary of non-respondent characteristics in sampling frame recorded y (≥95%). 1 score
b) Unsatisfactory recruitment rate, no summary data on non-respondents. Unsatisfactory recruitment rate, no summary data on non-respondents (<95%). 0 score
c) No information provided. 0 score

4) Ascertainment of the exposure (risk factor):

a) Validated measurement tool. 2 scores
b) Non-validated measurement tool, but the tool is available or described. 1 scores
c) No description of the measurement tool. 0 scores

Comparability: (Maximum 2 stars)

1) The subjects in different outcome groups are comparable, based on the study design or analysis. Confounding factors are controlled.

a) Data/results adjusted for relevant predictors/risk factors/confounders for example age, sex, time since vaccination, etc. 2 scores
b) Data/results not adjusted for all relevant confounders/risk factors/information not provided. 0 scores

Outcome: (Maximum 3 stars)

1) Assessment of the outcome:

a) Independent blind assessment using objective validated laboratory methods. 2 scores
b) Unblended assessment using objective validated laboratory methods. 2 scores
c) Used non-standard or non-validated laboratory methods with gold standard. 1 scores
d) No description/non-standard laboratory methods used. 0 scores

2) Statistical test:

a) The statistical test used to analyze the data is clearly described and appropriate, and the measurement of the association is presented, including confidence intervals and the probability level (p value). 1 scores
b) The statistical test is not appropriate, not described or incomplete. 0 scores
